# Stirred tank bioreactor process for chikungunya vaccine candidate VEEV-ΔC-CHIKV

**DOI:** 10.1371/journal.pone.0344564

**Published:** 2026-03-30

**Authors:** Tong Chang, ChengLin Deng, LiXia Xie, YiFan Zheng, YaNan Zhang, JiaWei Liu, ZhenFang Fu, Bo Zhang, Qi An, DaYong Tian

**Affiliations:** 1 R&D Department, Shanghai King-Cell Biotechnology Co., Ltd., Shanghai, Shanghai, China; 2 Key Laboratory of Virology and Biosafety, Wuhan Institute of Virology, Chinese Academy of Sciences, Wuhan, Hubei, China; National Taiwan Ocean University, TAIWAN

## Abstract

Chikungunya virus (CHIKV), a mosquito-borne *alphavirus* with significant public health impact, has caused recurrent epidemics across Africa, Asia, and the Americas. Despite the recent approval of a live-attenuated vaccine (Chix), scalable manufacturing processes for CHIKV vaccines remain critical to meet global demand. In this context, we previously constructed a chimeric virus, VEEV-ΔC-CHIKV, by replacing the backbone of ΔC-CHIKV with that of the VEEV replicon while retaining the CHIKV antigenic glycoprotein (E3-E2-6K-E1). A series of experiments have demonstrated that the chimeric virus exhibits safety, stability, and immunogenicity, making it a promising candidate for a new attenuated live vaccine strain. Therefore, to further validate its viral properties and explore the potential for large-scale production, we developed an optimized bioreactor-based production process for VEEV-ΔC-CHIKV. Utilizing adherent Vero cells in mechanically stirred bioreactors, we systematically optimized key parameters, including carrier type selection, cell seeding density, human albumin supplementation, and nutrient modulation, to maximize viral titers while minimizing the accumulation of metabolic byproducts. Results indicated that Cytodex™ 1 microcarriers outperformed Fibra-Cel carriers, enabling 10 consecutive harvests with viral titers ≥ 10^5^ PFU/mL (compared to 4 harvests for Fibra-Cel). When the initial cell density was 6.5 × 10^5^ cells/mL, the total virus yield was 77% higher than at a cell density of 2.5 × 10^5^ cells/mL. Adding 1.0% human albumin doubled total virus production compared to the addition of 0.5%. Nutrient modulation through the maintenance of glucose (>3 g/L) and glutamine (>0.5 g/L) in fed-batch systems enhanced peak titers to 10^6.4^ PFU/mL, representing a 2.2-fold increase compared to non-fed controls. This study establishes a scalable and cost-effective bioreactor platform for the production of the CHIKV vaccine, highlighting the critical interplay between nutrient optimization and viral yield enhancement, while emphasizing the advantages of automated process control in bioreactors to facilitate large-scale, high-yield viral production.

## 1. Introduction

Chikungunya virus (CHIKV), a member of the *Alphavirus* genus within the *Togaviridae family* [1], is an RNA enveloped virus with a diameter of approximately 70 nm. It consists of three structural proteins (capsid protein C, envelope proteins E1 and E2) and four non-structural proteins (nsP1, nsP2, nsP3, and nsP4). The viral genome is a single-stranded, non-segmented, positive-sense RNA that spans approximately 11–12 kb. CHIKV is primarily transmitted by Aedes mosquitoes, particularly in tropical and subtropical regions [2,3].

Humans are generally susceptible to CHIKV infection, which can manifest as either symptomatic or asymptomatic. The disease caused by CHIKV typically manifests as an acute febrile illness accompanied by joint pain, which can persist for weeks to years in many cases [1,4,5]. In newly emerged or epidemic areas, individuals of all age groups are susceptible; however, in endemic regions such as Africa and Southeast Asia, some symptoms in children infected with CHIKV are more severe [6,7]. CHIKV exhibits a global distribution but is predominantly endemic in Africa, South America, and Southeast Asia [8,9]. Furthermore, it has caused large-scale outbreaks in the Indian Ocean region in recent years. Since 2004, CHIKV has infected millions of people across Africa and Asia [5]. Between 2005 and 2007, massive outbreaks were reported in parts of Southeast Asia and neighboring islands, and cases emerged in Europe in 2007 [10,11]. It is estimated that half of the global population is at risk of infection [12]. According to the latest outbreak data on CHIKV released by the World Health Organization (WHO) and the European Centre for Disease Prevention and Control (ECDC), as of December 2024, 119 countries and territories have reported local transmission. However, high attack rates and ongoing susceptibility increase the risk of large-scale human infections. Additionally, due to limitations in surveillance and diagnostics, the potential for re-emergence remains high in previously affected areas [13]. From January to July 2025, approximately 240,000 CHIKV cases and 90 CHIKV-related deaths have been reported in 16 countries/ territories. Cases have been reported in the Americas, Africa, Asia, and Europe [14].

Large-scale CHIKV outbreaks can severely impact local socio-economic and public health systems, causing public panic and unease due to the virus’s high infectivity and rapid transmission, and overwhelming healthcare resources with a large number of patients. Currently, two Chikungunya vaccines are available in the United States. One is called Ixchiq, developed by Valneva SE, has been approved for marketing in several countries. This vaccine is a live-attenuated vaccine, carries a deletion of a large portion of the gene encoding nsP3 [15], has demonstrated good safety and immunogenicity [16,17], as single intramuscular injection can prevent CHIKV infection. Another candidate is Vimkunya®, a virus-like particle (VLP) vaccine developed by Bavarian Nordic. This vaccine received U.S. Food and Drug Administration (FDA) licensure in February 2025, followed by approval of the Advisory Committee on Immunization Practices (ACIP) recommendations for its use among U.S. travelers and laboratory personnel in April 2025 [18]. Several other CHIKV vaccines are in clinical or preclinical stages of development [16,19–23]. In our prior research, we engineered a live attenuated CHIKV vaccine candidate, namely VEEV-ΔC-CHIKV. Specifically, we replaced the backbone of ΔC-CHIKV with the venezuelan equine encephalomyelitis virus (VEEV) replicon but retained CHIKV antigen glycoproteins (E3-E2-6K-E1) to generate the chimeric VEEV-ΔC-CHIKV. This chimeric virus could propagate efficiently in Vero cells, achieving viral titers of up to 5 × 10^6^ PFU/mL. A single dose of immunization of VEEV-ΔC-CHIKV in mice was found to induce a CHIKV-specific neutralizing antibody response, and protected the mice from CHIKV challenge [24]. The successful viral production in Vero cells, combined with the satisfactory immunogenic response and safety profile of VEEV-ΔC-CHIKV, indicates that this vaccine candidate warrants further investigation in preclinical studies.

With advancements in biopharmaceutical technology, large-scale virus cultivation techniques have matured, encompassing both cell factory-based and bioreactor-based cultivation processes. Both methods enable the cultivation of higher densities of cells in a smaller three-dimensional space, facilitating large-scale virus production. Among these, bioreactor processes have gradually become the mainstream for large-scale virus cultivation, primarily for the following reasons: Firstly, bioreactors are more conducive to enhancing virus titers. They automatically control parameters such as pH, dissolved oxygen (DO), temperature, and agitation speed in the tank. By combining the principle that carriers provide a larger attachment area for cells to increase cell density with techniques such as cell perfusion and batch feeding, the system can effectively manage nutrient addition and metabolite removal. This maintains optimal cell conditions and ultimately significantly increases virus titers. Studies on bioreactor production processes for viruses such as herpes simplex virus type 1 (HSV-1) and rabies virus (RABV) have shown that, compared to other cultivation methods, bioreactor processes can increase titers by 10- to 100-fold [25,26]. Secondly, bioreactors are more suitable for large-scale applications [26–28]. Existing mature bioreactors for adherent cells have reached reaction volumes of 2000 L [29, 30], satisfying the industrial-scale production needs of most viruses. Thirdly, bioreactors offer advantages for aseptic production control. The cell and virus culture processes are particularly vulnerable to contamination by bacteria or molds. However, bioreactors are equipped with advanced online cleaning-in-place and sterilization-in-place technologies, along with highly automated control systems, which significantly minimize the risk of exposure during the culture process and ultimately reduce the risk of microbial contamination.

The cell matrix is a fundamental component of vaccine production, and its characteristics directly affect the safety, efficacy, and scalability of the manufacturing process. Currently, the cell lines approved for vaccine production include various cell lines such as MRC-5, Vero, MDCK, and CHO [31–35]. Among these, Vero cells are the most well-established and widely used, playing a crucial role in the global vaccine industry. Vero cells are a continuous cell line that can be subcultured indefinitely and are sensitive to many viruses, making them an ideal host for viral vaccine development. Worldwide, Vero cells have been approved for the production of various vaccines, including those for rabies, poliomyelitis, Japanese encephalitis, rotavirus, dengue fever, and influenza [36,37].

Attachment-dependent cells require a surface to adhere to in order to grow in a reactor. The cell density is limited by the surface area of the carrier, which depends on the number of carriers used. Commonly, adherent cells are cultured on microcarriers or Fibra-Cel carriers. In microcarrier culture, microcarriers are added to the reactor and kept in suspension by continuous stirring, allowing cells to attach and grow on their surfaces. The primary commercial microcarrier currently used is Cytodex™ 1, which has a specific surface area of approximately 4400 cm²/g and is typically used at concentrations ranging from 1.5 to 10 g/L. While a higher microcarrier concentration can increase cell density, it also demands more nutrients and oxygen. Therefore, it is important to carefully select the appropriate carrier concentration for each specific production process, as a higher concentration does not always lead to better outcomes. In suspension culture, a lower stirring speed is generally employed to ensure uniform mixing and efficient nutrient and gas transfer while avoiding the detrimental effects of high shear stress caused by excessive rotational speeds [36]. Fibra-Cel carrier culture involves filling a fixed bed with a specific number of Fibra-Cel carriers to support adherent cell growth. Commonly used Fibra-Cel carriers have specific surface areas typically ranging from 1000 to 2000 cm²/g. Due to their lower specific surface area compared to microcarriers, higher carrier concentrations (generally 20–50 g/L) are required to provide sufficient surface area for cell attachment. In Fibra-Cel carrier cultures, perfusion is often used to supply nutrients and gases, with stirring aiding their transport. However, the high cell density in these cultures can rapidly deplete nutrients and produce large amounts of metabolites, which can inhibit cell growth. Therefore, the optimal Fibra-Cel carrier concentration should be determined based on the specific production process. In Fibra-Cel cultures, cells do not come into direct contact with the stirring paddle, resulting in lower shear forces [38]. However, the larger size of Fibra-Cel carriers can cause them to settle and aggregate, hindering nutrient transport and cell growth. To prevent this, a higher stirring speed is typically used to ensure even dispersion of the Fibra-Cel carriers in the culture medium, thereby improving nutrient and gas transfer.

This study initially confirmed through comparative research that the bioreactor process is the most advantageous production method for the CHIKV vaccine candidate strain VEEV-ΔC-CHIKV. Subsequently, parameters such as carrier type, cell density, human albumin concentration, and the addition amounts of glucose and glutamine were investigated. Ultimately, a pilot-scale bioreactor cultivation process for VEEV-ΔC-CHIKV was successfully developed.

## 2. Materials and methods

### 2.1. Virus titration

One day prior to the plaque assay, BHK-21 cells were seeded at a density of 1 × 10^5^ cells per well in 24-well plates and cultured overnight in a 37°C incubator. On the following day, when cell confluence reached 90–100%, the monolayer was prepared for the plaque assay. The virus was serially diluted tenfold with dilution 0 representing the undiluted virus stock. Specifically, 15 μL of the undiluted virus was mixed with 135 μL of 2% FBS-supplemented Dulbecco’s Modified Eagle Medium (DMEM) to achieve a −1 dilution, and this process was repeated until a dilution of −6 was obtained. The culture medium was then removed from the 24-well plates containing confluent cells, and each virus dilution was added, starting from the highest dilution (100 μL) to the lowest. The plates were incubated in a 37°C incubator for one hour, with gentle shaking every 15 minutes. Subsequently, the virus inoculum was aspirated from each well in order from the highest to the lowest dilution, and 1 mL of an overlay mixture, comprising 200 mL of 4% methylcellulose and 200 mL of 4% FBS plus 2 × DMEM, was added to each well before incubating at 37°C for 3–4 days. Once plaques became visible to the naked eye, the overlay medium was removed, and 1 mL of a fixing/staining solution containing 3.7% formaldehyde and 1% crystal violet was added to each well and processed at room temperature for at least 30 minutes. After thorough washing under running water and drying, plaques were counted in wells where they were clearly visible, with the number ranging between 10 and 100, to calculate the viral titer in plaque-forming units per milliliter (PFU/mL).

### 2.2. Cell lines and cell culture

A working seed bank of Vero cells (Kidney cells of African Green Monkey, ATCC® CCL-81™), originally obtained from the American Type Culture Collection (Manassas, VA, USA), was established by King-Cell Biotech. The cell culture medium consisted of DMEM (Gibco) supplemented with 8% fetal bovine serum (FBS) (Lanzhou Rongye). Cells were thawed and cultured in T25 flasks (Corning) at 37°C and 70% − 80% relative humidity without carbon dioxide. Passaging was conducted every 7 days, and upon achieving confluent monolayers, cells were dissociated into single-cell suspensions using 0.25% porcine trypsin and subsequently expanded in a cell factory or bioreactor.

The Cytodex™ 1 microcarriers were subjected to three sequential rinses with phosphate-buffered saline (PBS, pH 7.2) to remove residual manufacturing particulates. After rinsing, the microcarriers were loaded into the bioreactor vessel and sterilized by autoclaving. Prior to cell inoculation, the bioreactor underwent a 24–72 h pre-culture phase under standardized conditions to validate sterility, ensure system integrity, and calibrate the DO probes. Dissociated Vero cells were introduced into the bioreactor under continuous stirring to promote uniform contact between the cells and microcarriers. Subsequently, parameters such as stirring speed, DO, pH, and temperature were set and adjusted to formally initiate the culture. The Vero cells continuously contact and adhere to the surface of the microcarriers through stirring, allowing them to grow and expand across the entire surface. During this process, the cells remain tightly adhered to the microcarriers surfaces, while the microcarriers are suspended by stirring, resulting in the indirect suspension of the cells. It is important to note that Vero cells are not suspension cells; rather, they are adherent-dependent cells.

### 2.3. Virus

The VEEV-∆C-CHIKV virus was obtained from a previous study [24]. In that study, the chimeric virus VEEV-ΔC-CHIKV was engineered using reverse genetics with a pBR322-based plasmid system containing the infectious clone of the attenuated Venezuelan equine encephalitis virus strain TC83 (pACYC-VEEV-TC83). The native VEEV structural gene cassette was precisely replaced with the CHIKV envelope glycoprotein operon (E3-E2-6K-E1), resulting in a single-cycle replicon that retains the complete nonstructural replication machinery of VEEV but cannot produce infectious VEEV virions due to the absence of VEEV structural proteins. This construct supports robust RNA replication and exclusively displays CHIKV envelope glycoproteins on the surface of infected cells. Compared to our original ∆C-CHIKV [39], the VEEV-∆C-CHIKV, yielded viral titers as high as 5 × 10^6^ PFU/mL in Vero cells after extensive serial passages.

### 2.4. Bioreactor preparation

#### 2.4.1. Microcarrier-based bioreactor.

The control system consists of 5 L triple parallel bioreactors (BioPAS) with working volumes ranging from 2 L to 5 L. The bioreactor vessels were labeled R101, R201, and R301, respectively. Microcarriers were washed three times with 0.01M PBS 24 hours prior to the experiment and introduced into the bioreactor vessels at a concentration of 4 g/L (reaction volume). The vessels were then filled with 0.01M PBS to the specified volume. After calibrating the pH electrodes, the electrodes and silicone tubing were assembled onto the vessels, which were subsequently steam-sterilized. Following sterilization, the PBS was drained, and growth medium was added to the prescribed volume. The cultures were maintained at 37°C with stirring at 50 rpm for pre-cultivation. According to the standard operating procedures, the key parameters for the culture process are set as follows: temperature 34–38°C, stirring at 40–60 rpm, pH 7.0–7.4, and DO at 40%−60%.

#### 2.4.2. Fibra-Cel carrier-based bioreactor.

The control system comprised a 2 L triple parallel bioreactors (Tchuyee) with bioreactor vessels labeled 1#, 2#, and 3#. Twenty-four hours prior to the experiment, Fibra-Cel carriers were added to the vessels at a concentration of 30 g/L (reaction volume) and soaked overnight in 0.01M PBS. After draining, the carriers were rinsed thrice with 0.01M PBS. After pH electrode calibration, the electrodes and silicone tubing were assembled, and the vessels were steam-sterilized. Subsequently, the PBS was removed, and growth medium was added to the specified volume. The cultures were maintained at 37°C with stirring at 120 rpm for pre-cultivation. According to the standard operating procedures, the key parameters for the culture process were set as follows: temperature 34–38°C, stirring at 100–150 rpm, pH 7.0–7.4, and DO at 40%−60%.

### 2.5. Three cultivation processes for VEEV-ΔC-CHIKV

Vero cells, cultured to form a confluent monolayer, were dissociated to a density of 1.0 × 10^5^ cells/mL and inoculated into a T225 flask, a cell factory (1264 cm^2^), and a 2 L bioreactor. The cultures were maintained at 37°C for 7 days. During this period, cell growth was monitored, and the cell expansion fold and viability were calculated prior to be infected with the virus. Subsequently, the cells were infected with VEEV-ΔC-CHIKV at a multiplicity of infection (MOI) of 0.01. Samples were collected daily post-infection for VEEV-ΔC-CHIKV virus titration. In this study, Vero cells were stained with trypan blue, and calculated the cell viability by optical microscope [40].

### 2.6. Selection of carrier types for bioreactors

This study utilized microcarriers (Cytodex™ 1, Cytiva) and Fibra-Cel carriers (Disks, Growth), with bioreactor vessel working volumes of 2 L and initial cell seeding densities of 1.0 × 10^6^ cells/mL. After 7 days of cell culture, the cells were infected with VEEV-ΔC-CHIKV at an MOI of 0.01 and cultivated continuously. Daily samples were collected for virus titration, while glucose, glutamine, glutamate, and lactate levels in the bioreactor vessel solutions were monitored. Glucose and glutamine consumption, as well as oxygen consumption, were calculated. The parameters for the Fibra-Cel carrier bioreactor were set at 37°C, pH 7.2, DO at 50%, and stirring at 120 rpm. For the microcarrier bioreactor, the parameters were 37°C, pH 7.2, DO at 50%, and stirring at 40–60 rpm.

### 2.7. Selection of initial cell density

The control system utilized a 5 L triple parallel bioreactors (BioPAS), each equipped with a 5 L working volume vessel and microcarriers (Cytodex™ 1, Cytiva) for cell production. The initial cell density was set at 2.5 × 10^5^ cells/mL for vessel R101, while vessel R201 had an initial cell density of 6.5 × 10^5^ cells/mL. The bioreactors operated under the following parameters: 37°C, pH 7.2, DO at 50%, and stirring at 40–60 rpm. Cell perfusion was conducted from days 2–5, totaling 2.5 culture volumes. On day 6, the cells were washed five times with DMEM wash solution (Gibco) and subsequently infected with VEEV-ΔC-CHIKV at a MOI of 0.01. Virus harvesting began on day 3 post-infection, with a daily harvest volume equivalent to 0.5 of the working volume. Virus titration was measured, growth curves were plotted, and nutrient content and consumption were monitored.

### 2.8. Determination of human albumin concentration

The control system consisted of 5 L triple parallel bioreactors (BioPAS), each with a 5 L working volume bioreactor vessel and microcarriers (Cytodex™ 1, Cytiva) for production. Cell suspensions at a density of 2.5 × 10^5^ cells/mL were inoculated into two bioreactor vessels and cultivated under identical conditions for 7 days. Virus culture media containing human albumin (RongSheng, NMPA approved) at working concentrations of 1.0% and 0.5% were added to the two vessels, respectively, followed by infection with VEEV-ΔC-CHIKV at an MOI of 0.01. Daily samples were collected for virus titration, growth curves were plotted, and nutrient content and consumption were monitored.

### 2.9. Exploration of nutrient supplementation levels

The control system consisted of 2 L triple parallel bioreactors (BioPAS), each with a 2 L working volume and microcarriers (Cytodex™ 1, Cytiva) for production. Cell suspensions at a density of 2.5 × 10^5^ cells/mL were inoculated into two bioreactor vessels and cultivated under identical conditions for 7 days. Post-infection, vessel R101 employed a strategy of no glucose or glutamine supplementation, allowing natural virus growth and nutrient consumption. Harvesting began on day 2 post-infection, with a daily harvest volume of 0.5 culture volumes. In contrast, vessel R201 employed a strategy of supplementing glucose (China Shijiazhuang Pharmaceutical Group Co., Ltd.) and glutamine (Daesang Corporation) to maintain concentrations above 3 g/L and 0.5 g/L, respectively, with a daily harvest volume of 0.5 culture volumes. All other conditions were identical to those in the cell density comparison experiment. By examining the rate of cell detachment, monitoring nutrient content and consumption, measuring viral titer, and assessing the number of harvests, we aimed to determine which feeding strategy was more effective. The cell detachment rate is operationally defined as the proportion of microcarrier surface area vacated by detached cells relative to the total available surface area. Specifically, prior to viral infection, cells formed a confluent monolayer covering the entire microcarrier surface. Post-infection, virus-induced cytopathic effects triggered progressive cell detachment, which was estimated by calculating the ratio of vacated surface area (derived from detached cells) to the total microcarrier surface area.

### 2.10. Ethics statements

This study was designed as an exploratory preliminary investigation, focusing on validating the basic feasibility of the new process. Before the study began, all authors were informed orally and provided their consent to conduct this research. This study did not involve animals, animal tissues, or animal-derived materials. It also did not involve human subjects, human tissues, or human-derived materials. All experiments were conducted in accordance with relevant institutional and national guidelines for research without the involvement of animals or humans. This study complies with the principles expressed in the Declaration of Helsinki. Moreover, this research meets the ethical exemption requirements outlined in the “Ethical Review Measures for Life Sciences and Medical Research Involving Humans” promulgated by China, and can be exempted from ethical review.

## 3. Results

### 3.1. Bioreactor technology demonstrates superior suitability for the production of the vaccine candidate strain VEEV-ΔC-CHIKV

**Fig 1:**
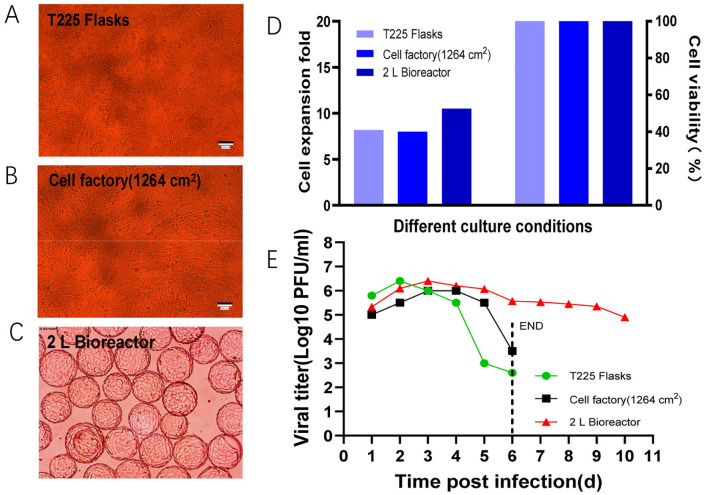
Comparison of VEEV-ΔC-CHIKV growth characteristics under different culture conditions. **(A)** Growth status of Vero cells in T225 flask. **(B)** Growth status of Vero cells in cell factory (1264 cm^2^). **(C)** Growth status of Vero cells in 2 L bioreactor. **(D)** Cell expansion fold and cell viability of Vero cells in T225 flask, cell factory (1264 cm^2^), and 2 L bioreactor. **(E)** Growth curves of VEEV-ΔC-CHIKV virus in T225 flask, cell factory (1264 cm^2^), and 2 L bioreactor.

To identify the most optimal pilot-scale production process for VEEV-ΔC-CHIKV, the virus was cultivated in T225 flasks, a cell factory (1264 cm^2^), and a 2 L bioreactor utilizing microcarriers. Prior to virus inoculation, Vero cells exhibited a plump morphology with no free-floating cells across all three culture conditions (Figs 1A-1C). The viability of Vero cells remained above 96% for all culture methods. Notably, the cell proliferation rate was significantly higher in the bioreactor, achieving a 10-fold increase compared to the other two culture conditions (Fig 1D). The multi-step growth curves of VEEV-ΔC-CHIKV under the three culture conditions revealed that viral titers increased during the first two days post-inoculation (dpi). Specifically, the viral titers in the T225 culture condition reached their peak earliest and were the highest, attaining a maximum of 10^6.4^ PFU/mL on 2 dpi, followed by a rapid decline. On 5 dpi, the viral titer in the cell factory culture system also exhibited a rapid decline. In contrast, the viral titer in the bioreactor culture system reached its peak on 3 dpi and subsequently declined at a slower rate, maintaining a level of 10^4.9^ PFU/mL by 10 dpi (Fig 1E) (S1–S4 Tables).

### 3.2. Microcarriers are more suitable than Fibra-Cel carriers for the production of VEEV-ΔC-CHIKV in bioreactor

**Fig 2:**
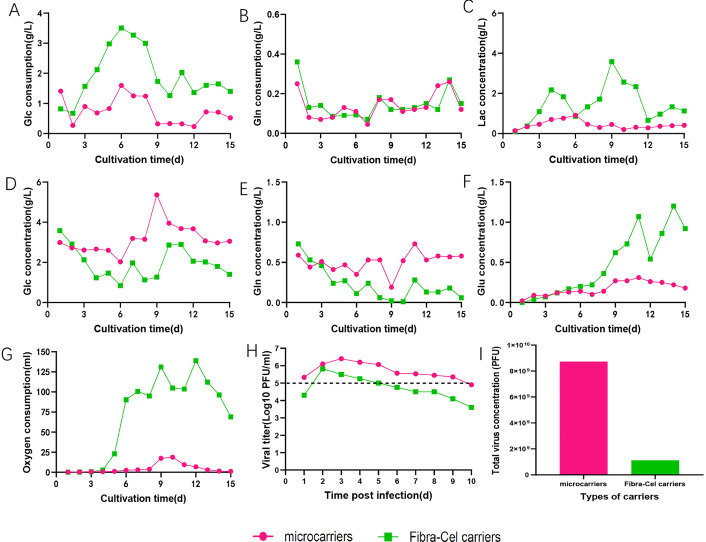
Comparison of VEEV-ΔC-CHIKV cultivation processes using microcarriers and Fibra-Cel carriers bioreactors. **(A)** Glucose consumption. **(B)** Glutamine consumption. **(C)** Lactate concentration in the bioreactor. **(D)** Glucose concentration in the bioreactor. **(E)** Glutamine concentration in the bioreacor. **(F)** Glutamate concentration in the bioreactor. **(G)** Oxygen consumption. **(H)** Virus titer under both carriers. **(I)** Total virus concentration obtained under both carriers. Glucose, Glc; Glutamine, Gln; Glutamate, Glu; Lactate, Lac.

To identify the most suitable carrier type for the bioreactor process of VEEV-ΔC-CHIKV, two 2 L bioreactor vessels were utilized, employing microsphere and Fibra-Cel carriers, respectively. The growth curves, nutrient consumption characteristics, and metabolite production properties of VEEV-ΔC-CHIKV were investigated in these two vessels. As illustrated in Fig 2A and 2G, starting from 3 and 5 dpi, the glucose and oxygen consumption in the Fibra-Cel carrier bioreactor vessel were significantly higher than in the microsphere carrier bioreactor vessel. By the conclusion of the production process, total glucose and oxygen consumption in the Fibra-Cel carriers bioreactor vessel were 2.5-fold and 15-fold higher, respectively, than those observed in the microsphere carrier bioreactor vessel. During the entire duration of virus cultivation, there was no significant difference in glutamine consumption between the two carrier culture system (Fig 2B). Beginning on 6 dpi, the concentrations of lactate and glutamate in the culture medium of the Fibra-Cel carriers bioreactor vessel were markedly higher than those in the microsphere carrier bioreactor (Fig 2C and 2F). The multi-step growth curve of VEEV-ΔC-CHIKV, as illustrated in Fig 2H, indicates that under the microsphere carrier bioreactor condition, the virus could be harvested 10 times consecutively with a viral titer of no less than 10^5^ PFU/mL, whereas under the Fibra-Cel carrier culture condition, the virus could only be harvested 4 times. Finally, the total virus concentration under the microcarriers culture mode was 7.8 -fold greater than that under the Fibra-Cel carriers (Fig 2I). It is important to note that the levels of glucose and glutamine in the culture medium were consumed too rapidly during the cultivation process in the Fibra-Cel carriers reactor. As a result, the remaining quantities were insufficient to meet the normal growth requirements of the cells. Therefore, small additional amounts of glucose and glutamine were added daily.

### 3.3. A cell density of 6.5 × 10^5^ cells/mL is favorable for achieving higher viral titers of VEEV-ΔC-CHIKV

**Fig 3:**
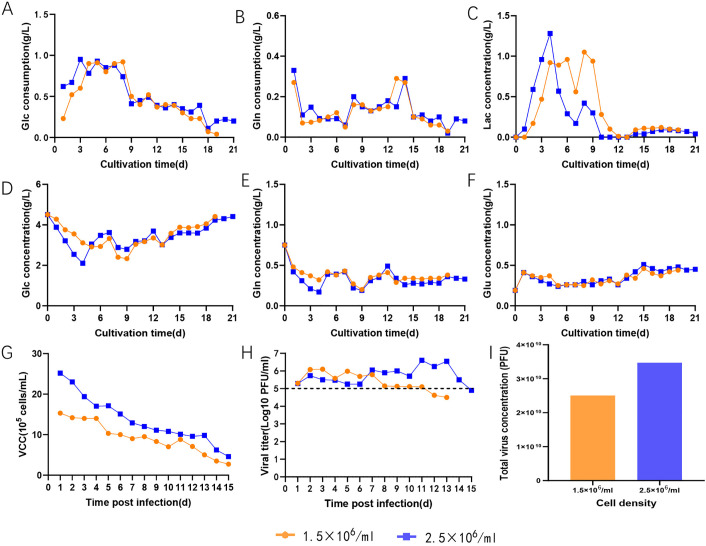
Investigation of different cell density conditions. **(A)** Glucose consumption. **(B)** Glutamine consumption. **(C)** Lactate concentration in the bioreactor. **(D)** Glucose concentration in the bioreactor. **(E)** Glutamine concentration in the bioreacor. **(F)** Glutamate concentration in the bioreactor. **(G)** Viable cell density in the bioreactor. **(H)** Virus titer at two cell densities. **(I)** Total virus concentration obtained at the two cell densities. Glucose, Glc; Glutamine, Gln; Glutamate, Glu; Lactate, Lac.

To investigate the impact of cell density on the viral titer of VEEV-ΔC-CHIKV in the microsphere carrier bioreactor, inoculations were performed using initial cell densities of 2.5 × 10^5^ cells/mL and 6.5 × 10^5^ cells/mL. Throughout the cultivation process, differences in cell density did not significantly affect the consumption of various nutrients or the concentrations of metabolites (Fig 3A-3F). On 6 dpi, the cell densities reached 1.5 × 10^6^ cells/mL and 2.5 × 10^6^ cells/mL, respectively (Fig 3G). The growth curve of VEEV-ΔC-CHIKV revealed that viral titers were significantly higher under the high-cell-density condition compared to the low-cell-density condition at 7 dpi, and the viral titers remained at high levels for a longer duration (Fig 3H). The total virus concentration under the high-cell-density culture condition was 77% higher than that under the low-cell-density condition (Fig 3I).

### 3.4. A concentration of 1% human albumin is more conducive to the amplification of VEEV-ΔC-CHIKV

**Fig 4:**
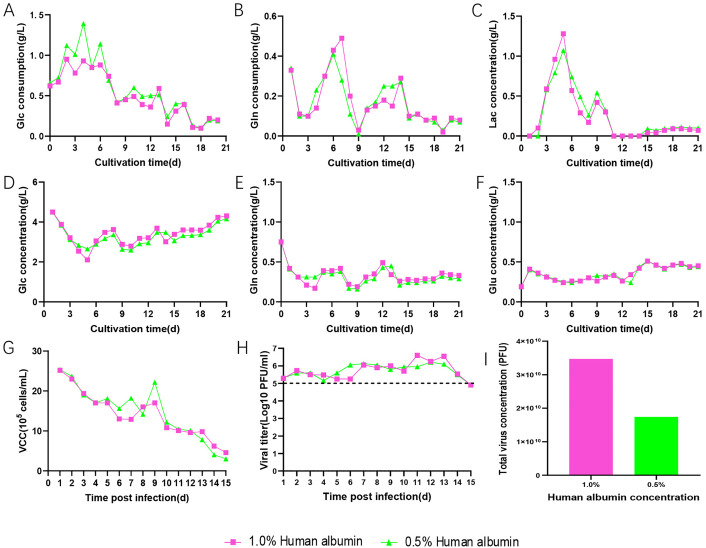
Investigation of different human albumin concentrations. **(A)** Glucose consumption. **(B)** Glutamine consumption. **(C)** Lactate concentration in the bioreactor. **(D)** Glucose concentration in the bioreactor. **(E)** Glutamine concentration in the bioreacor. **(F)** Glutamate concentration in the bioreactor. **(G)** Viable cell density in the bioreactor. **(H)** Virus titer at the two human serum albumin concentrations. **(I)** Total virus concentration obtained at two human serum albumin concentrations. Glucose, Glc; Glutamine, Gln; Glutamate, Glu; Lactate, Lac.

To investigate the minimum concentration of human albumin required to maintain high-titer production of VEEV-ΔC-CHIKV, virus culture media containing 1% and 0.5% human albumin were used during the virus cultivation phase. Throughout the entire cultivation process, variations in human albumin concentration did not significantly affect the consumption rates of various nutrients or the levels of metabolites (Fig 4A-4F). The viral growth curves (Fig 4H) showed similar growth patterns and harvest frequencies for VEEV-ΔC-CHIKV under both human albumin concentrations. However, when 1% human albumin was added, the total virus concentration of VEEV-ΔC-CHIKV was 2-fold higher than that add 0.5% human albumin (Fig 4I).

### 3.5. It is essential to maintain a glucose concentration of 3 g/L and a glutamine concentration of 0.5 g/L in the medium to enhance the viral titer of the VEEV-ΔC-CHIKV

**Fig 5:**
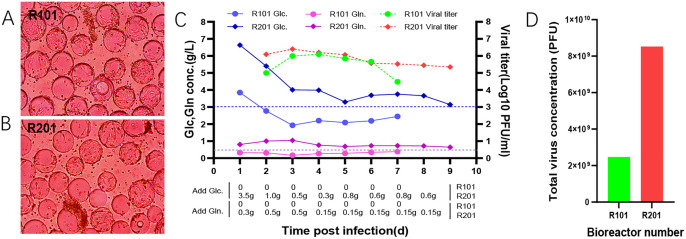
Investigation of different feed strategies. **(A)** Cell growth status in the R101 bioreactor. **(B)** Cell growth status in the R201 bioreactor. **(C)** Overview of glucose and glutamine concentrations, virus titer, and amounts of glucose and glutamine added in both bioreactors after virus inoculation. **(D)** Total virus concentration obtained from both bioreactors.

To investigate the direct relationship between the cultivation of VEEV-ΔC-CHIKV and nutrient supply, two identical bioreactor vessels were employed for cell cultivation. Following the inoculation of VEEV-ΔC-CHIKV, one bioreactor vessel implemented a no-feed strategy, with only basal medium being supplemented, while the other bioreactor vessel implemented a feed strategy, with additional glucose and glutamine being added to maintain glucose concentrations above 3 g/L and glutamine concentrations above 0.5 g/L in the culture medium. Glucose and glutamine concentrations in both bioreactors were monitored daily, and samples were collected to measure the viral titer of VEEV-ΔC-CHIKV. The cell status and detachment rates in the two bioreactors were similar after the inoculation of VEEV-ΔC-CHIKV, with cell detachment rates exceeding 90% by 7 dpi (Fig 5A and 5B). Growth curves showed that the viral titer in the fed-batch bioreactor reached a peak of 10^6.4^ PFU/mL on 3 dpi and could be harvested 8 times with a viral titer of no less than 10^5^ PFU/mL in contrast, the viral titer in the no-feed bioreactor reached a peak of 10^6.1^ PFU/mL on 4 dpi and could only be harvested 5 times with a viral titer of no less than 10^5^ PFU/mL (Fig 5C). The total virus yield in the fed-batch bioreactor was 2.2-fold higher than that in the no-feed bioreactor (Fig 5D).

## 4. Discussion

In both carrier culture modes, the culture temperature was maintained at 37°C, the DO level was set to 50%, and the pH was controlled at 7.2. These conditions were based on the general culture characteristics of Vero cells, as supported by extensive literature and research. However, the stirring rates differed between the two carrier modes. The stirring speed balances efficient mass transfer of nutrients without causing excessive shear stress to the carrier structure. Specifically, the Fibra-Cel carrier reactor was set to 100–150 rpm, while the microcarrier reactor operated at 40–60 rpm. This difference is primarily due to the larger size of Fibra-Cel carriers, which can cause them to settle and aggregate, hindering nutrient transport and cell growth. To prevent this, a higher stirring speed is typically used to ensure even dispersion of Fibra-Cel carriers in the culture medium, thereby improving nutrient and gas transfer.

In our comparative assessment of cell culture carriers for vaccine production, we evaluated Fibra-Cel carriers (30 g/L loading) against microcarriers (4 g/L loading). The Fibra-Cel system provided a 3.4-fold higher specific surface area (120,000 cm² vs. 35,200 cm²), theoretically enabling superior cell adhesion density for enhanced viral replication. However, contrary to our expectations, VEEV-ΔC-CHIKV titers in Fibra-Cel cultures (peak: 10^5.5^ PFU/mL) underperformed compared to microcarrier cultures (peak: 10^6.4^ PFU/mL), despite the addition of glucose and glutamine to the Fibra-Cel cultures in the bioreactor. Metabolic profiling revealed that the dense framework of Fibra-Cel led to the depletion of glucose and glutamine, while accelerating the accumulation of lactate, glutamate, and ammonium ions. According to the evidence presented, while the Fibra-Cel carrier reactor provides Vero cells with a larger attachment area and more nutrients, it also results in faster cell proliferation and a higher nutrient demand. This leads to greater metabolic waste production, ultimately creating a metabolically stressed microenvironment that compromises both cell viability and viral productivity. This may be the primary reason for the unsatisfactory virus titer. These findings indicate the limitations of over-relying on surface area metrics when designing experimental protocols for adhesion bioreactors and emphasize the importance of maintaining nutritional balance and implementing effective metabolic control strategies.

Human albumin serves multiple roles in the development of virus-based vaccines. It functions as a chelator of heavy metals, a regulator of pH and osmotic pressure, a carrier of growth factors, a surfactant, and a vital nutrient in cell culture [41–43]. Human albumin also influences virus stability; various viruses, including Coxsackie virus B3, are particularly sensitive to the presence of serum albumin, and human albumin can enhance virus stability in cell culture [44]. Some mammalian cells are more susceptible to physical stress in bioreactor environment [45]. Acting as a physical shear protectant [43], human albumin plays an important role in protecting these mammalian cells from damage in sparged and airlift-type bioreactors [46]. Recombinant human albumin is particularly well-suited for these applications because it meets stringent regulatory requirements for clinical use [47]. However, due to the high production costs and limited supply stability of human albumin, it is imperative to systematically investigate and determine the optimal concentration for its use in process development, balancing process implementation with cost control to the greatest extent. In the future, it is necessary to explore additional alternatives. For example, biological materials produced through recombinant technology can be used to replace those of animal origin, or chemical materials can be used as substitutes for biological materials, to improve quality control, ensure a stable supply, and reduce costs.

Mammalian cells are extensively used in vaccine manufacturing. Viral replication is highly dependent on the host cellular nutritional metabolism [48]. Glucose and glutamine are considered the primary energy suppliers and carbon sources for cell growth [48,49]. Viruses rely on the glucose metabolism of host cells to obtain the necessary energy and biosynthetic precursors. Additionally, glucose helps maintain metabolic homeostasis both inside and outside the cell, preventing the accumulation of harmful metabolites. Glutamine serves as a vital nitrogen source in cell culture and is particularly critical for the synthesis of viral proteins and nucleic acids [50,51]. Supplementing with glutamine can partially rescue viral replication [52]. However, this process also produces metabolic waste. The breakdown of glucose generates lactate, which is released into the culture medium, resulting in a decrease in pH and adversely affecting the cellular environment [53,54]. The breakdown of glutamine produces ammonium ions, to which many cells are sensitive, inhibiting their growth even at low concentrations. Sensitivity to ammonium ions varies significantly among different cell lines. Studies have shown that glucose and glutamine are nearly completely consumed during the growth phase of MDCK cells [55]. Similarly, transfected S2 cells (S2AcRVGP) consume glucose and glutamine as primary substrates during their rapid growth phase [56]. This pattern of nutrient consumption aligns with our findings and supports the general principle that viruses replicate more efficiently at adequate cell density and under optimal cellular conditions. Some viruses entry into the infectious phase is also accompanied by increased consumption of glucose and glutamine; for example, herpes simplex virus infection increases the rate of glycolysis [57], while vaccinia virus infection leads to enhanced glutamine uptake [58]. Additionally, PRRSV infection promotes both glucose and glutamine uptake [59]. Therefore, glucose and glutamine play central roles in cell and virus culture within bioreactors, not only providing essential energy and material foundations but also directly influencing cell viability through complex metabolic pathways, which in turn affects viral replication efficiency. Proper regulation of the supply of these two nutrients can significantly enhance virus harvest titers, increase harvest frequencies, and improve overall virus yield. In this study, we demonstrated that maintaining glucose concentrations above 3 g/mL and glutamine concentrations above 0.5 g/mL in the culture medium resulted in higher titers of the VEEV-ΔC-CHIKV virus and greater total virus harvest yields compared to production conditions with lower glucose and glutamine concentrations. In our future research, we plan to explore more effective supplementation strategies for glucose and glutamine and to investigate the optimal maintenance concentrations of these nutrients in virus culture media.

The bioreactor microcarrier cell culture system is currently one of the most widely used methods for vaccine production. To meet the increasing global demand for vaccines, the bioreactor expansion culture process is becoming increasingly stringent. First, to increase cell density, the number of microcarriers is raised; however, an excessive number of cells can lead to insufficient stirring and sedimentation of microcarriers. Conversely, increasing the stirring speed to prevent cell sedimentation can cause shear stress damage to the cells due to the stirring paddle. Excessive cell density combined with low agitation speed results in inadequate oxygen and nutrient delivery, which adversely affects cell health and product quality [36]. Therefore, it is essential to balance shear stress and medium transfer as effectively as possible. Second, the stepwise amplification culture methods used in microcarrier cell culture systems primarily include trypsin digestion amplification and sphere-to-sphere amplification processes [60]. Both methods, however, cause cell loss, reduce cell viability, and negatively impact amplification efficiency. To control vaccine costs and pricing, upstream vaccine production must prioritize two key objectives: maximizing cell yield and maintaining optimal cellular physiological activity. These measures directly enhance the efficiency of virus production.

With the global spread of chikungunya, developing an effective vaccine can further prevent the disease’s transmission. The vaccine candidate VEEV-ΔC-CHIKV described in this study employs a chimeric replicon strategy across viral genera by replacing the ΔC-CHIKV backbone with the replicon of VEEV while retaining the key CHIKV antigen glycoproteins (E3-E2-6K-E1). In recent years, studies have reported long-term health effects in some individuals vaccinated with the chimpanzee adenovirus-vectored COVID-19 vaccine, which has been associated with an increased risk of COVID-19 infection. These cases suggest that the safety design of viral vectors should be a core consideration in vaccine development, particularly regarding the persistent immune response or potential risks caused by the vector itself. The VEEV replicator-based CHIKV vaccine strategy described here offers a valuable technical approach to address these concerns. This vaccine ensures immunogenicity by incorporating VEEV replicative elements to enable efficient antigen expression, allowing it to function effectively as a vaccine. At the same time, the disease-causing genes of wild-type CHIKV are removed through backbone replacement. The VEEV replicon can only complete antigen protein synthesis within the host cell and cannot assemble into a complete infectious virus particle. This mechanistic design avoids traditional viral vector risks such as vector replication and wild-type virus back mutation. This approach achieves an optimal balance between safety and immunogenicity, offering a novel strategy for CHIKV vaccine development. Additionally, this study is the first to develop a bioreactor process for this novel vaccine candidate. Comparative experiments have demonstrated that the bioreactor process is the most effective production method for this vaccine. It overcomes the limitations of traditional cell factory production and establishes a new paradigm for process development, similar to approaches used for chimeric viruses. Finally, by optimizing system parameters, a pilot-scale bioreactor process for VEEV-ΔC-CHIKV was successfully developed, laying the foundation for subsequent large-scale bioreactor production.

## 5. Conclusions

In this study, a series of experiments were conducted to establish an optimized bioreactor-based platform for the scalable production of the chimeric live-attenuated CHIKV vaccine candidate VEEV-ΔC-CHIKV. This experiment is a preliminary exploratory study, the experimental design was centered on demonstrating feasibility and optimizing critical parameters within a single, highly controlled system. which were conducted as single-group trials without parallel control groups, which we fully acknowledge as a limitation of this research. Future studies will aim to perform in-depth optimization of cultivation strategies, metabolic regulation of nutrient balance, and scaled-up bioreactor processes to further advance the development of VEEV-ΔC-CHIKV (S1 File).

## Supporting information

S1 File Chart of experimental design. (PDF)

S1 Table Comparison with T225 flasks, 2 layer cell factories and bioreactors. (DOCX)

S2 Table The composition of cell growth culture medium. (DOCX)

S3 Table The composition of virus propagation culture medium. (DOCX)

S4 Table The full spellings of all the abbreviations used in the article. (DOCX)

S1 Data The values used to build graphs. (XLSX)
