# Supplementary figures and images for: Stirred tank bioreactor process for chikungunya vaccine candidate VEEV-ΔC-CHIKV
Source: PLoS One. 2026 Mar 30;21(3):e0344564. doi: 10.1371/journal.pone.0344564 (PMC13035149; doi:10.1371/journal.pone.0344564)

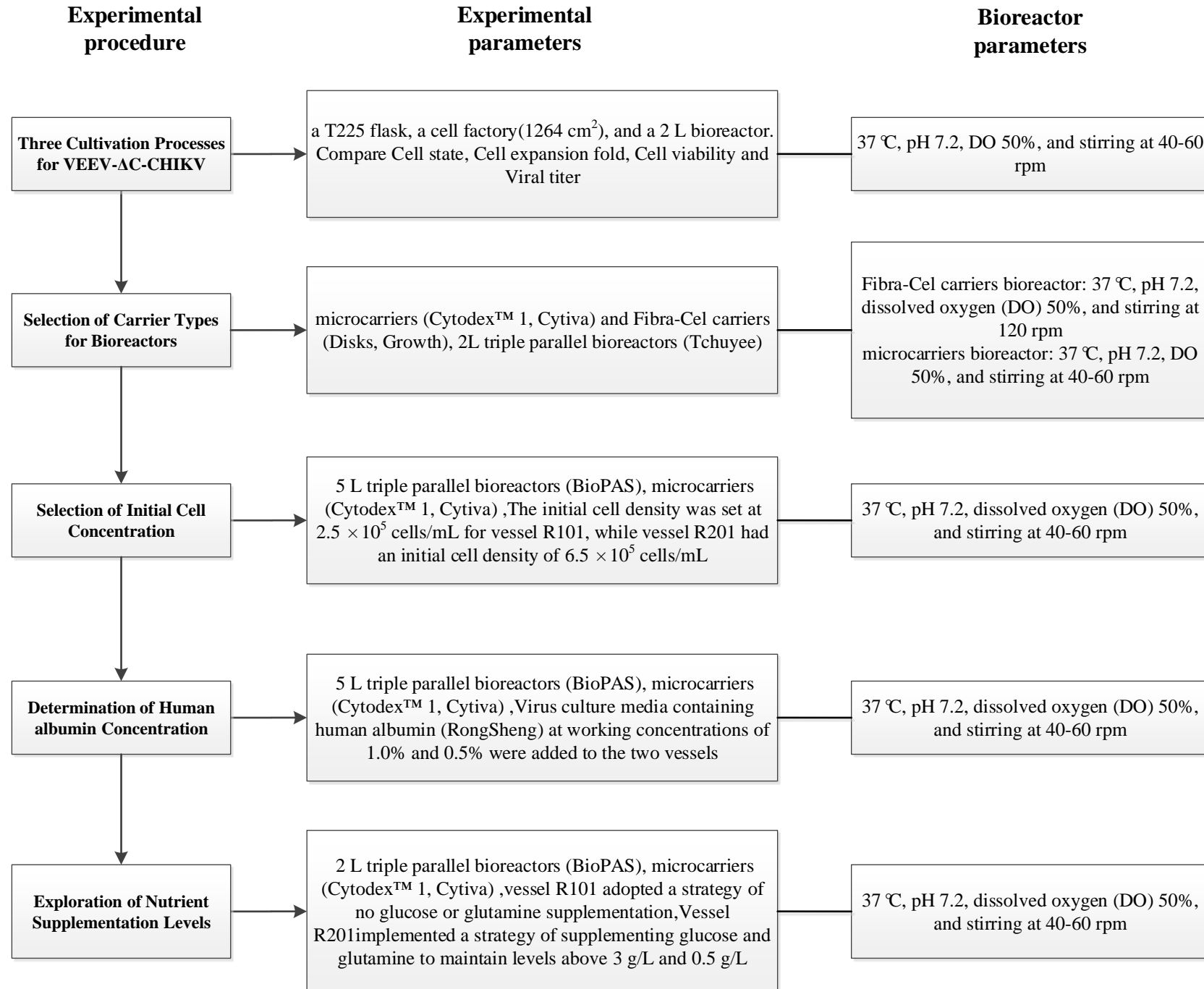

Supplement: S1 File — (PDF) [file pone.0344564.s001.pdf]
