# Supplementary material for: Stirred tank bioreactor process for chikungunya vaccine candidate VEEV-ΔC-CHIKV
Source: PLoS One. 2026 Mar 30;21(3):e0344564. doi: 10.1371/journal.pone.0344564 (PMC13035149; doi:10.1371/journal.pone.0344564)
Supplement: S1 Table — (DOCX) [file pone.0344564.s002.docx]

S1 Table. Comparison with T225 flasks, 2 layer cell factories and bioreactors.

|  | T225 flasks | 2 layer cell factory | 2L bioreactor/ microcarriers | 2L bioreactor/  Fibra-Cel carriers | 5L bioreactor/ microcarriers |
| --- | --- | --- | --- | --- | --- |
| Proportion of carriers | - | - | 4 g/L | 30 g/L | 4 g/L |
| Available surface/ cm2 | 225 | 1,264 | 35,200 | 120,000 | 88,000 |
| Working volume/ L | 0.09 | 0.4 | 2 | 2 | 5 |
| Initial cell seeding densities / × 105 cells/mL | 1.0 | 1.0 | 1.0-10.0 | 10.0 | 2.5-6.5 |
| Applicability | lab-scale | lab-scale | pilot-scale | pilot-scale | pilot-scale |
| Automatic control | no | no | yes | yes | yes |
| Shear force | no | no | higher | lower | higher |
| Amplification potential | lower | lower | higher | higher | higher |
