# Supplementary material for: Stirred tank bioreactor process for chikungunya vaccine candidate VEEV-ΔC-CHIKV
Source: PLoS One. 2026 Mar 30;21(3):e0344564. doi: 10.1371/journal.pone.0344564 (PMC13035149; doi:10.1371/journal.pone.0344564)
Supplement: S3 Table — (DOCX) [file pone.0344564.s004.docx]

S3 Table. The composition of virus propagation culture medium.

| Component | volume ratio | volume (ml/10L) |
| --- | --- | --- |
| DMEM | 97% | 9700 |
| 3% L-glutamine solution | 1% | 100 |
| 7.5% sodium bicarbonate solution | 1% | 100 |
| human albumin | 1% | 100 |
