# Supplementary material for: Stirred tank bioreactor process for chikungunya vaccine candidate VEEV-ΔC-CHIKV
Source: PLoS One. 2026 Mar 30;21(3):e0344564. doi: 10.1371/journal.pone.0344564 (PMC13035149; doi:10.1371/journal.pone.0344564)
Supplement: S4 Table — (DOCX) [file pone.0344564.s005.docx]

S4 Table. The full spellings of all the abbreviations used in the article.

| Abbreviation | Full Spelling |
| --- | --- |
| CHIKV | Chikungunya virus |
| VEEV | Venezuelan equine encephalomyelitis virus |
| FDA | Food and Drug Administration |
| ACIP | Advisory Committee on Immunization Practices |
| DO | Dissolved Oxygen |
| HSV-1 | herpes simplex virus type 1 |
| RABV | rabies virus |
| CIP | cleaning-in-place |
| SIP | sterilization-in-place |
| DMEM | Dulbecco's Modified Eagle Medium |
| FBS | fetal bovine serum |
| NMPA | National Medical Products Administration |
| MOI | multiplicity of infection |
| IACUC | Institutional Animal Care and Use Committee |
| WHO | World Health Organization |
| ECDC | European Centre for Disease Prevention and Control |
| dpi | days post inoculation |
| MDCK | Madin-Darby Canine Kidney |
| PRRSV | Porcine reproductive and respiratory syndrome virus |
